# The Significance of *VDR* Genetic Polymorphisms in the Etiology of Preeclampsia in Pregnant Polish Women

**DOI:** 10.3390/diagnostics11091698

**Published:** 2021-09-17

**Authors:** Justyna Magiełda-Stola, Grażyna Kurzawińska, Marcin Ożarowski, Tomasz M. Karpiński, Krzysztof Drews, Agnieszka Seremak-Mrozikiewicz

**Affiliations:** 1 Division of Perinatology and Women’s Disease, Poznan University of Medical Sciences, Polna 33, 60-535 Poznan, Poland; justyna.magielda@gmail.com (J.M.-S.); gene@gpsk.am.poznan.pl (G.K.); kdrews@gpsk.am.poznan.pl (K.D.); asm@data.pl (A.S.-M.); 2 Department of Biotechnology, Institute of Natural Fibres and Medicinal Plants, Wojska Polskiego 71b, 60-630 Poznan, Poland; 3 Chair and Department of Medical Microbiology, Poznan University of Medical Sciences, Wieniawskiego 3, 61-712 Poznan, Poland; tkarpin@ump.edu.pl; 4 Laboratory of Molecular Biology in Division of Perinatology and Women’s Diseases, Poznan University of Medical Sciences, Polna 33, 60-535 Poznan, Poland

**Keywords:** *VDR* genetic polymorphisms, preeclampsia, molecular biology methods

## Abstract

For the first time in the Polish population, we aimed to investigate associations between the *VDR* gene single-nucleotide polymorphisms (SNPs) *BsmI* (rs15444410), *ApaI* (rs7975232), *FokI* (rs19735810), and *TaqI* (rs731236) and the development of preeclampsia (PE). A case–control study surveyed 122 preeclamptic and 184 normotensive pregnant women. The polymerase chain reaction–restriction fragment length polymorphism (PCR-RFLP) method was performed to examine the maternal *VDR FokI*, *BsmI*, *TaqI*, and *ApaI* polymorphisms. The *VDR BsmI* *AA* homozygous genotype was statistically significantly more frequent in preeclamptic women compared to the control group (*p* = 0.0263), which was also associated with a 2-fold increased risk of PE (OR = 2.06, *p* = 0.012). A correlation between the *VDR BsmI* polymorphism with systolic and diastolic blood hypertension was noted. Furthermore, 3-marker haplotype *CTA* (*TaqI/ApaI/BsmI*) was associated with significantly higher systolic (*p* = 0.0075) and diastolic (*p* = 0.0072) blood pressure. Association and haplotype analysis indicated that the *VDR BsmI A* allele could play a significant role in the PE pathomechanism and hence could be a risk factor for PE development in pregnant Polish women. These results indicate the importance of the *VDR BsmI* polymorphism and reveal that this variant is closely associated with a higher predisposition to hypertension.

## 1. Introduction

Preeclampsia (PE) (ICD-10 code 014), a specific condition during pregnancy, still remains a significant clinical problem in perinatal medicine. Hypertensive disorders occur in about 5–10% of pregnancies throughout the world, and preeclampsia complicates the course of 3% of pregnancies in industrialized countries [1]. In Poland, as in other regions of the world, the frequency of this serious complication is estimated at an incidence rate of 2–3% [2,3]. Preeclampsia is connected to a significant risk of the appearance of serious complications in mothers and fetuses, which may require labor induction or a preterm caesarean section. Additionally, preeclampsia could be associated with an increased risk of several disorders in mothers later in life, such as cardiovascular diseases, chronic arterial hypertension, stroke, metabolic syndrome, and chronic kidney disease. Infants born to women with preeclampsia are at increased risk of, first of all, cardiovascular diseases and hypertension, but also insulin resistance, diabetes, and even neurodevelopmental disorders [4,5,6]. Thus, considering the above-mentioned, it undoubtedly becomes clear that it is worth paying a significant amount of attention to research into the pathogenesis of preeclampsia.

PE is characterized by maternal hypertension and proteinuria with or without pathological edema [7]. The etiology of PE is multifactorial; an important role is played by factors related to vasoconstriction and remodeling of the placenta, coagulation disorders, oxidative stress, lipid metabolism disorders, impaired adaptation of the immune system, and implantation disorders [8]. Thus far, a direct association between the above mechanisms and the risk of preeclampsia has been not established. Studies suggest that the placenta is central to the pathogenesis of PE [9,10]. According to Jena [10], a drug delivery system targeting the placenta needs to be developed for the potential treatment of PE. There are over 40 metabolites of vitamin D in peripheral blood, but it is believed that vitamin D status in the human is best reflected by the concentration of its 25-monohydroxylated compound isoform 25(OH)D, calcidiol, which is the most frequently measured metabolite [11]. In humans, the minimum normal concentration of the 25(OH)D metabolite is 30 ng/mL. However, this form is not biologically active and must be hydroxylated by 1 α-hydroxylase in the kidneys to its 1,25(OH)2D form [12,13,14].

This situation is more complicated in pregnant women in which the vitamin D metabolism remains complex and not fully understood. It has been documented that the concentration of ionized calcium and 25(OH)D in pregnant women does not change, and the concentration of PTH remains on the lower limit. The placental transport of vitamin D metabolites in human is unknown. It was shown that in deciduae and syncytiotrophoblasts metabolites 1,25(OH)2D and 24,25(OH)2D are both synthesized. On the other hand, it was also revealed that the concentration of the 1,25(OH)2D metabolite begins to increase from early pregnancy and achieves twice of its initial value by the third trimester. This is the result of the synthesis of VD metabolites in the maternal kidney, but also in the decidua, placenta, and fetal kidneys (1-alpha-hydroxylase activity has been demonstrated in all these tissues) [15,16,17]. An increase in the level of the 1,25(OH)2D metabolite enables a significant increase in intestinal calcium absorption, especially in the third trimester of pregnancy. Additionally, in the third trimester of pregnancy, the action of the active form 1,25(OH)2D metabolite allows the production of calcium from the bones, which is manifested by an increase in the concentration of bone turnover markers in pregnant women. It seems that the action of the active 1,25(OH)2D form, especially in the third trimester of pregnancy, is the key factor in ensuring proper vitamin D and calcium metabolism in the mother and fetus [18,19].

Vascular endothelial function is considered as a marker in many cardiovascular problems, including preeclampsia, which is a disease of the maternal endothelium [20,21]. In normal pregnancies, the endothelium is known to regulate vascular tone by balancing vasoconstriction with vasodilation to provide adequate perfusion pressure to target organs [22]. However, during preeclampsia there is a change in the endometrial levels of various angiogenic growth factors, such as vascular endothelial growth factor A (VEGF-A); mRNAs encoding VEGF-C; placental growth factor (PlGF); the angiopoietins, angiopoietin 1(Ang1) and Ang2; and the receptors VEGFR-3 (Flt-4), Tie 1, and Tie 2 [23]. Moreover, during preeclampsia a very low level of nitric oxide production leads to impaired vasodilatation. The progress of endothelial disfunction is dependent on the development of an inflammatory process [24,25]. At this point it should be emphasized that preeclampsia has been associated with low calcitriol levels, and vitamin D deficiency is correlated with a higher risk of the development of this disease [26]. It is well known that vitamin D metabolites influence the development of physiological pregnancy through various mechanisms. It participates in the process of the transformation of endometrial cells into decidual cells [27], promotes the invasion of human trophoblasts in vitro [28], and increases expression of the *HOXA10* gene, which is necessary for implantation in early pregnancy [29]. The active 1,25(OH)2D also affects the proper functioning of endothelial cells, demonstrating a vasoprotective effect. In this role, this metabolite regulates the activity of NO synthase, enhances the activity of antioxidant enzymes such as superoxide dismutase, and neutralizes the activity of NADPH, an enzyme involved in the formation of free oxygen radicals [30].

According to Barrera [31], VDR mediates the effect of calcitriol, which dose-dependently inhibited IL-10 expression in normal and preeclamptic pregnancies. Xu et al. [32] showed that downregulation of VDR expression contributes to increased endothelial inflammatory response in preeclampsia and is associated with upregulation of the expression of VCAM-1 in systemic vessel endothelium in this disease. Xu et al. [32] also revealed that VDR expression has been significantly reduced in cells treated with TNFα. Moreover, it was shown that upregulation of miR-126 expression induced by 1,25(OH)_2_D_3_ is mediated through VDR. Thus, this upregulation of miR-126-3p expression may be involved in a mechanism of the anti-inflammatory activity of vitamin D.

The active 1,25(OH)2D form regulates blood pressure by influencing endothelial smooth muscle proliferation [33]. Vitamin D also affects the functioning of the renin–angiotensin–aldosterone system (RAAS) by inhibiting the transcription of renin, a proteolytic enzyme that raises blood pressure. Mice experimentally lacking the *VDR* gene developed arterial hypertension independent of classical regulatory mechanisms such as plasma volume and osmolality [34]. Recent studies have shown that impaired placement causes disturbed expression of the placental renin–angiotensin system, which may lead to systemic disturbances in the functioning of the RAAS system and the development of PE [30]. Vitamin D also modulates the functioning of the immune system, affecting the balance of Th1/Th2 helper lymphocytes, and in this way, it reduces the secretion of pro-inflammatory cytokines and increases the secretion of anti-inflammatory cytokines [35]. This leads to a state of transient immunosuppression necessary for the proper development of pregnancy at an early stage. Given the variety of functions, it is no wonder that disturbances in vitamin D homeostasis lead to many disorders, and vitamin D deficiency is also listed among the risk factors for the development of preeclampsia. It has been shown that pregnant women whose vitamin 25 (OH)D level falls below 20 ng/mL have an increased risk of developing preeclampsia [36].

VDR is a transcription factor that, when bound to the retinoid X receptor, binds to vitamin D responsive elements (VDREs) in the DNA promoter sequence of many genes. Vitamin D acts on cells after binding to a specific steroidal vitamin D receptor (VDR). In normal pregnancy, VDR expression was demonstrated in trophoblasts and temporal villi, in the smooth muscles of the placental vessels, in the nuclei of the fetal villi stromal cells, and in the nuclei of fetal epithelial cells [8]. The *VDR* gene, cloned for the first time by Baker et al. [37], is located on chromosome 12 (12q11-q13). The most intensively studied *VDR* polymorphisms are *FokI*, *TaqI*, *BsmI*, and *ApaI* variants. Three single nucleotide polymorphisms (*ApaI*, *BsmI* in intron 8 section, and *TaqI* in exon 9 (*Ile352Ile*) are in strong linkage disequilibrium (LD) with each other and additionally with a poly(A) length polymorphism (rs17878969) in the 3′ untranslated region of the *VDR* gene. These variants do not change the amino acid sequence in the VDR protein but can influence the gene expression through regulation of mRNA stability [38]. The most frequently studied variant that results in altered structure of the VDR protein is the *FokI* variant causing a change of *C>T* nucleotides (*ACG*-*ATG*) in exon 2. In carriers of the *C* allele (designated *F*) the VDR protein is three amino acids shorter (424 amino acids) and more active. In contrast, individuals carrying the *T* allele (designated *f*) synthesize a less active, full-length VDR protein (427 amino acids) [39].

Previously, no relationship between PE susceptibility and the maternal and placental *VDR BsmI* rs1544410, *TaqI* rs731236, and *ApaI* rs7975232 polymorphisms has been observed [40]. However, it was revealed that the maternal and placental *VDR FokI* polymorphism has been associated with lower PE risk in the dominant model (*Ff* + *ff* vs. *FF*) and it was concluded that these genotypes could decrease PE risk [40].

In addition to the above, it is worth noting that the results of the meta-analysis of 11 observational studies showed the relationship between the *VDR* polymorphism (*FokI*—seven, *BsmI*—eight, *ApaI*—five, and *TaqI*—seven studies) and the risk of preterm birth, and it was shown mainly the protective effect of the *BsmI* polymorphism against the risk of preterm birth in terms of the allele (*A* vs. *G*: OR = 0.74; 95% CI 0.59–0.93) and in the recessive model (*AA* vs. *GG* + *AG*: OR = 0.62; 95% CI 0.43–0.89) [41].

Considering the important role of vitamin D and its receptor in the development of physiological pregnancy and the possible influence of vitamin D deficiency on the occurrence of preeclampsia, it can be assumed that VDR gene polymorphisms influence the development of preeclampsia. In the present study, the four vitamin D receptor gene SNPs *FokI* (rs2228570), *BsmI* (rs1544410), *ApaI* (rs7975232), and *TaqI* (rs731236) were analyzed to assess their potential value for risk prediction in preeclamptic women.

## 2. Materials and Methods

### 2.1. Subjects

A total of 306 Caucasian women of Polish origin (122 with preeclampsia and 184 heathy normotensive pregnant women) were enrolled in the study. All patients were recruited from the Division of Perinatology and Women’s Diseases, Poznan University of Medical Sciences. Preeclampsia was diagnosed according to American College of Obstetricians and Gynecologists (ACOG) criteria [42]. The exclusion criteria were as follows: age younger than 19 or older than 35, diabetes mellitus, hemolytic disease, habitual smokers, multifetal pregnancy, chronic hypertension, endocrinological diseases, kidney diseases, or obesity. Depending on the time of disorder appearance, preeclampsia is classified either as early onset (EOPE), which requires delivery before 34 weeks’ gestation, or late-onset (LOPE), with delivery at or after 34 weeks.

In each woman with preeclampsia, venous blood was taken for laboratory tests leading to preeclampsia diagnosis routinely after their admission to hospital. The laboratory tests such as urea, uremic acid, total protein, creatinine, liver enzymes ALT, AST, and maternal proteinuria were performed in III trimester of gestation. In the laboratory analysis of the above tests, the protocol methods used routinely in our hospital were applied.

The participants in the control group were in the third trimester of a healthy pregnancy and had to be normotensive, without any fetal disorder, pathological states, or multiple pregnancies. All patients were Caucasians of Polish origin. The enrolled women were informed in detail about the study and provided their written consent to collect venous blood samples for diagnostic and scientific purposes including genotyping. The study was approved by the Local Bioethical Committee at Poznan University of Medical Sciences (No: 1129/18).

### 2.2. Genotyping

The preliminary data on the selected VDR gene SNPs are shown in Table 1. Genomic DNA was extracted from blood samples stored in EDTA-coated tubes using the QIAamp DNA Mini Kit according to the manufacturer’s instructions (Qiagen, Hilden Germany). The concentration of genomic DNA was quantified using a NanoDrop Spectrophotometer (Thermo Scientific, Waltham, MA, USA). Genotyping of the *VDR* gene polymorphisms was performed in the Molecular Biology Laboratory of Poznan Medical Science University by polymerase chain reaction and restriction fragment length polymorphism (PCR-RFLP). The rs7975232 and rs731236 polymorphic sites of the *VDR* gene were analyzed by a method described in the study of Pani et al. [43]. A 740-bp PCR product was digested with *ApaI* (Eurx, Gdańsk, Poland) at 25 °C overnight. The same PCR product was also digested overnight with the restriction enzyme *TaqI* (Eurx, Gdańsk, Poland) at 65 °C. The 825-bp fragment encompassing the rs1544410 polymorphic site in intron 8 was amplified using primers and PCR conditions described by Morrison et al. (1994) [44]. Mva12691 (*BsmI*) (Thermo Scientific, Waltham, MA, USA) enzymes were added directly to the PCR products and digested at 37 °C. The rs2228570 was identified using the method described by Harris et al. [45]. The products of PCR amplification were cleaved with the restriction enzyme *FokI* (Eurx, Gdańsk, Poland) at 37 °C overnight. DNA fragments obtained after respective restrictive enzyme digestion and the DNA size marker were electrophoresed on a 2.5% agarose gel with Midori Green Advanced DNA Stain (Nippon Genetics, Europe GmbH, Düren, Germany). For documentation of results, gel pictures were taken under ultraviolet light.

### 2.3. Statistical Analysis

Statistical analyses were conducted using R version 4.0.3 version (R Foundation for Statistical Computing, Vienna, Austria, accessed on 20 October 2020) [46]. For continuous variables, normality was checked by the Shapiro–Wilk test. Continuous variables normally distributed were expressed as mean ± SD and in the absence of normal distribution as median and interquartile range (IQR). Categorical variables were expressed as numbers or percentages. Clinical characteristics between groups were compared using Student’s *t*-test or Mann–Whitney U test. Pearson’s χ^2^ and the Fischer test for nominal variables was used. For each SNP, the Hardy–Weinberg equilibrium (HWE) was assessed using Pearson’s goodness-of-fit χ^2^ statistic. Differences in allele and genotype frequencies between the case and control subjects, odds ratios (ORs) and associated 95% confidence intervals (95% CIs) were evaluated using the SNPassoc package for R [47]. Two-tailed values of *p* < 0.05 were accepted to be statistically significant. The Bonferroni multiple-comparison correction method was employed to calculate the corrected *p* value. The general linear model (GLM) was applied to compare blood pressure levels between genotypes and haplotypes.

The haplotype-based association analysis was performed using Haploview version 4.2 software ((Cambridge, MA, USA, https://www.broadinstitute.org/haploview/haploview (accessed on 1 October 2020)). Linkage disequilibrium (LD) parameters r^2^ and Dʹ and haplotype frequencies were calculated. Distribution of haplotypes was compared in case and control groups with chi-squared tests. Significant *p* values were corrected using the 10,000-fold permutation test.

## 3. Results

### 3.1. Baseline Demographics and Clinical Characteristics of Study Population

The preeclamptic women and controls had similar mean age (30.12 ± 5.52 vs. 30.58 ± 4.41 years, respectively, *p* = 0.4507). In the PE group lower gestational age, neonatal birth weight and Apgar score in the first and fifth minute, as well as higher systolic/diastolic blood pressure and pre-/post-pregnancy BMI index compared to the control group (all, *p* < 0.001), were noted. Accordingly, pre-pregnancy BMI and primiparity were selected as a covariate to be controlled for in subsequent analysis. In preeclamptic women delivery by caesarean section was more frequent (90.16 vs. 33.15% in controls, *p* < 0.001), and more women in this group were primiparas (61.48 vs. 39.13% in controls, *p* = 0.0001). Clinical characteristics of the study population are summarized in Table 2.

In the PE group early onset preeclampsia was diagnosed in 57 women (46.72%) and 65 (53.28%) cases were diagnosed as late-onset. Comparing early and late-onset groups, the most interesting observation was lower gestational age in the early onset subgroup (30.60 vs. 36.54 weeks, *p* < 0.001) and systolic blood pressure (175.65 vs. 166.97 mmHg, *p* = 0.0171). The two subgroups also differed in neonatal birthweight and Apgar score in the first and fifth minute (all, *p* < 0.001). For the PE group also blood chemistry tests were performed. In the early onset group a statistically significantly lower level of total protein (5.47 vs. 5.81 g/dL in LOPE, *p* = 0.0089) and significantly higher levels of ALT (53.83 vs. 23.08 in LOPE, *p* = 0.0411) and AST (52.17 vs. 25.50 in LOPE, *p* = 0.0492) were observed. The distribution of selected clinical and laboratory parameters in PE cases and controls is shown in Table 3.

### 3.2. Associations between SNPs and Preeclampsia Risk

The genotyping completion rates were 100% and all SNPs conformed to HWE in the control group (*p* > 0.05). The genotype distribution of *BsmI* rs1544410 differs significantly from that expected by HWE only in the preeclamptic group (*p* = 0.021). Higher minor allele frequency (MAF) for *BsmI* rs1544410 was found in PE cases (0.467 vs. 0.353 in controls, OR = 1.61, 95% CI = 1.15–2.23, *p* = 0.005) and this association remained significant after applying correction for multiple testing (*p* = 0.020). Additionally, for *TaqI* rs731236, the *C* allele was associated with increased risk of PE (OR = 1.42, 95% CI: 1.02–1.99, *p* = 0.038), but the association was not significant after Bonferroni correction (Table 4).

The risk of PE associated with a specific *VDR* genotype was confirmed by testing the effect of pre-pregnancy BMI and primiparity as confounding factors. Significantly higher frequencies of homozygous *BsmI AA* genotype carriers were found between PE cases and control women, respectively 27.0 vs. 15.2%, *p* = 0.0263 (recessive model: OR = 0.48, 95% CI = 0.27–0.85, *p* = 0.0119). This association remained significant after adjusting for maternal BMI (*p_adj._* = 0.0113, recessive model *p_adj._* = 0.0048).

The dominant models for rs1544410 (*BsmI*) and rs7975232 (*ApaI*) were significantly associated with PE after adjusting (*p_adj._* = 0.0334 and *p_adj._* = 0.0425, respectively). The prevalence of the maternal VDR rs731236 (*TaqI*) *TC* and *CC* genotype were higher in preeclamptic group than the healthy pregnant women. When the mode of inheritance was dominant, the OR (95% CI) for *TT* vs. *TC + CC* was 0.63 (0.39–1.00, *p* = 0.0498, *p_adj._* = 0.0375). The distribution of VDR genotypes in PE women and controls is summarized in Table 5. The prevalence of the maternal *VDR* rs7975232 *GG* genotype was higher in the healthy pregnant women than the preeclamptic group (25.5 vs. 18.9%). When the mode of inheritance was dominant, the OR (95% CI) for *TT* vs. *TG* + *GG* was 1.63 (0.97–2.74, *p* = 0.0660, *p_adj._* = 0.0503).

In the next stage a stratified analysis was performed. Women with preeclampsia were divided into two subgroups: early onset preeclampsia (57 cases), and late-onset preeclampsia (65 cases). Relative to the controls, significantly increased risk of EOPE was found to be associated with the *TT* genotype of *ApaI* (rs7975232) polymorphism in a codominant model, compared with *GG* genotype (*p*= 0.0724, OR = 2.85, 95%CI = 1.08–7.57, *p_adj._* = 0.036). Compared with *GG* genotype, markedly increased risk of preeclampsia was associated with the *TT + TG* genotypes in a recessive model (*p* = 0.0275, OR = 2.45, 95% CI = 1.04–5.78, *p_adj._* = 0.0185). No significant association was observed between *ApaI* polymorphism and risk of late-onset PE (*p* > 0.05). Significantly increased risk of early onset preeclampsia was also identified to be associated with the *A* allele of the *BsmI* (rs1544410) polymorphism (*p* = 0.0451 in the dominant model and *p_adj._* = 0.0305 in the recessive model). For this variant a difference was also found between late-onset preeclampsia and controls in the recessive model (*p* = 0.0165, OR = 0.43, 95% CI = 0.22–0.85). For the *FokI* (rs2228570) polymorphism, the only statistically significant difference was observed between the EOPE and control groups in the recessive model after adjusting for BMI (*p_adj._* = 0.0402). Our data indicated no significant difference in the allele frequencies of *TaqI* (rs731236) and *FokI* (rs2228570) polymorphisms between early and late-onset preeclampsia compared to the control group (Table 6).

### 3.3. Association between VDR Gene Variants and Blood Pressure

Linear regression analysis was used to examine genotype associations with blood pressure for all 306 women (Table 7). One SNP, rs1544410 (*BsmI*), was found to be associated with both systolic blood pressure (SBP) and diastolic blood pressure (DBP) in all genetic models evaluated. This association remained significant after adjusting for maternal BMI and primiparity (SBP *p*-value 0.0033, 0.0172 and 0.0015, DBP *p*-value 0.0026, 0.0073 and 0.0020 in the codominant, dominant, and recessive model, respectively). Carriers of the *A* allele in rs1544410 showed higher blood pressure in both SBP and DBP than non-carriers (*GG* genotype). The minor allele *C* of *TaqI* SNP (rs73123) increased the SBP and DBP of women who carried them in the dominant model, and the minor allele *G* of *ApaI* SNP (rs7975232) had the opposite effect on SBP and DBP.

When the examined women were divided into subgroups, statistically significant differences we observed for diastolic blood pressure in the codominant model and *BsmI* (*p_adj._* = 0.0431) and *TaqI* (*p_adj._* = 0.0469) variants in the controls. In the group of early onset preeclampsia, the differences were noted for *TaqI* in the recessive (for SBP *p_adj._* = 0.0301 and for DBP *p_adj._* = 0.0033) and the codominant model for DBP (*p_adj._* = 0.0130). For the late onset PE group, the *FokI* polymorphism and systolic blood pressure significant difference in recessive model was obtained (*p_adj._* = 0.0182).

### 3.4. Linkage Disequilibrium and Haplotype Analysis of VDR SNPs

**Figure 1:**
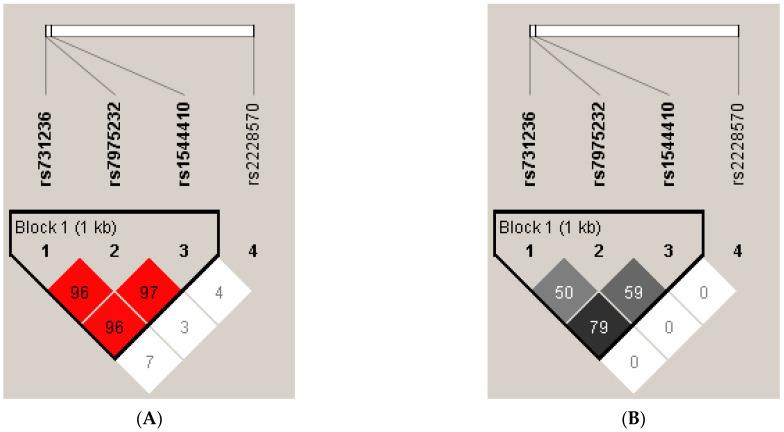
Linkage disequilibrium pattern between the four SNPs of the *VDR* gene determined by Haploview software (**A**)—Lewontin’s D′ on the left and (**B**)—r2 on the right.

It was also investigated whether the four SNPs were in linkage disequilibrium. A schematic diagram of the LD pattern among genotyped SNPs is shown in Figure 1 (the range of the area surrounded by lines indicates that three SNPs of the *VDR* gene were contained in a haplotype and in a state of linkage disequilibrium). Block 1 comprised rs1544410 (*BsmI*), rs7975232 (*ApaI*) and rs731236 (*TaqI*), which were in strong pairwise LD (D’ > 0.95; r^2^ > 0.5). However, rs2228570 (*FokI*) was poorly correlated with the other three SNPs.

The most common haplotypes of the study polymorphisms, calculated by Haploview 4.2, are summarized in Table 8. Eight four-marker haplotypes with a frequency of more than 1% were detected (*TGGC* 25.2%, *TGGT* 22.5%, *CTAC* 19.0%, *CTAT* 16.6%, *TTGT* 7.3%, *TTGC* 4.4%, *TTAT* 2.2%, and *TTAC* 1.6%). In the haplotype analysis, only the *TTAT* haplotype occurrence was more frequent in the study group compared to the control group (0.036 vs. 0.013, *p* = 0.0572). The other four-locus haplotypes did not show any difference in frequencies between the cases and controls. For three -marker haplotype analyses, *CTA* (*p* = 0.0241) and *TAT* (*p* = 0.0509) were more frequent in preeclamptic women, while the *TGG* haplotype was more common in the uncomplicated pregnancy group (0.510 vs. 0.425, *p* = 0.0376). In the permutation testing, none of the significant haplotypes was significantly associated with PE.

In a haplotype analysis the 3 -marker haplotype *CTA* (*TaqI/ApaI/BsmI*) was associated with a significantly higher systolic (*p* = 0.0075, 95%CI = 2.36–15.33) and diastolic (*p* = 0.0072, 95%CI = 1.50–9.56) blood pressure risk as compared with the most frequent haplotype *TGG* (Table 9).

## 4. Discussion

Polymorphic variants of vitamin D are involved in many biological processes and in the etiology of several disorders, including cardiovascular diseases, diabetes, osteoporosis, and cancer. These observations were supported by epidemiological studies. The association of genetic VDR polymorphisms with these disorders were indicated by recent studies for *BsmI*, *ApaI*, and *TaqI* nonfunctional variants. These variants probably could be in linkage disequilibrium (LD) with other functional polymorphisms, which explains the results observed recently. The explanation of this relationship may be helpful in defining the group of patients predisposed to development of the disorders as well as in creating tailored therapy [37,48].

It has often been indicated that some *VDR* gene variants could be associated with an increased risk of chronic hypertension [49,50]. Thus far, several publications have analyzed the relationship of *VDR* gene polymorphisms with the occurrence of certain complications in pregnancy [27]. It has been suggested that *VDR* polymorphism could be associated with the occurrence of gestational diabetes [51,52,53], preterm labor [54], recurrent miscarriages [55,56], gestational cholestasis [40], and intrauterine fetal restriction that often coexisted with PE and placental insufficiency [27]. Thus far, however, no unequivocal results have been obtained determining the importance of *VDR* gene variants in the pathways influencing the placenta function, regulating implantation, hormone secretion, or immune modulation.

According to our best knowledge, there have been no studies evaluating the frequency of *VDR* gene polymorphisms in the Polish population of pregnant women with preeclampsia that have been published. In our study, the most interesting observation was the demonstration that the *VDR* *BsmI* *AA* homozygous genotype was statistically significantly more frequent in preeclamptic women compared to the control group (*p* = 0.0263), which was also associated with a 2-fold increased risk of PE (OR = 2.06, *p* = 0.012). Additionally, the frequency of the *VDR* *BsmI* *A* allele was significantly higher in the PE group compared to controls (*p* = 0.005). The association between PE and *VDR BsmI* polymorphism occurrence persisted even after adjustment for the pre-pregnancy BMI value as a predisposing factor for hypertension in pregnancy. Moreover, the stratification analysis, after dividing the PE group into early onset and late-onset forms, showed a relationship between the EOPE form and *VDR BsmI A* allele carriage in both dominant (*p* = 0.0451) and recessive (*p_adj._* = 0.0441) models, as well as between the LOPE form and *VDR BsmI A* allele carriage in the recessive model (*p* = 0.0165).

Additionally, we noted a higher frequency of the homozygous *VDR TaqI CC* genotype (*p* = 0.117) and the mutated *VDR TaqI C* allele (*p* = 0.038) in the PE group than in the controls. However, stratified analysis did not show any significant association between *VDR TaqI* polymorphism and EOPE/LOPE.

Our analysis did not show a statistically significant difference for either the *VDR ApaI* or *FokI* polymorphism between the whole PE group and the controls. However, for these polymorphisms we noted an association between possession of the *VDR ApaI T* allele and EOPE in the codominant model (*p* = 0.0431) and in the recessive model (*p* = 0.0275), as well as the *VDR FokI T* allele in the recessive model after BMI adjustment (*p_adj._* = 0.0402).

The studies considering the participation of VDR gene variants in populations of pregnant women with preeclampsia in various regions of the world show inconclusive results. Farajian-Mashhadi et al., in a group of pregnant women from Iran (152 PE/160 healthy pregnant women), observed a lower frequency of the heterozygous *VDR FokI Ff* genotype in women with PE, both in maternal serum (*p* = 0.02) and in the placenta (*p* = 0.06). The *VDR FokI* polymorphism was also correlated with a lower risk of PE in the dominant model (*Ff* + *ff* vs. *FF*) both in the mother and in the placenta, indicating that both genotypes, heterozygous *Ff* (OR = 0.5, *p* = 0.007) and homozygous *ff* (OR = 0.5, *p* = 0.02), may reduce the risk of PE. No relationship was found between the presence of PE and *VDR BsmI, TaqI*, and *ApaI* polymorphisms in maternal serum and placenta. Additionally, no relationship was detected between the studied *VDR* polymorphisms and the severity of PE [57].

The influence of *FokI, TaqI*, and *BsmI* polymorphisms of the *VDR* receptor and the level of vitamin D (25(OH)-D) on the risk of PE and blood pressure were analyzed in the study by Rezavand et al. (100 PE/100 healthy pregnant women). The mean level of vitamin D in women with PE was significantly lower (16.6 vs. 19.6 ng/mL in healthy pregnant women, *p* < 0.001). A significantly higher frequency of the *VDR FokI C* allele was observed in women with PE (83%) compared to the control group (74%), which was associated with a 1.72-fold higher risk of PE. In the whole group of women, systolic and diastolic blood pressure values were significantly higher in carriers of the *VDR FokI CC* genotype compared to carriers of the *VDR FokI TC* and *TT* + *TC* genotypes. Moreover, *VDR TaqI* and *VDR BsmI* variants were not associated with the risk of PE. The study showed a relationship between the *VDR FokI* polymorphism, as well as insufficient level of vitamin 25(OH)-D in the mother’s serum, and the risk of developing PE. Additionally, the influence of an insufficient level of vitamin 25(OH)-D and the *VDR FokI* polymorphism on increased blood pressure in female carriers of variants of this polymorphism was also demonstrated [58].

The study by Zhan et al. analyzed a group of pregnant women from the Chinese population (Han province, 402 PE/554 healthy pregnant women in the third trimester). The relationship between three polymorphisms of the *VDR* gene (*VDR FokI* rs2228570, Cdx2 rs11568820 and *BsmI* rs1544410) and the risk of PE appearance was analyzed. A statistically significant difference was observed in the frequency of the *VDR FokI (G/A)* polymorphism genotypes between the PE group and the control group (*p* = 0.001). The *VDR G* allele was a risk factor for the development of PE (*p* = 0.002, OR = 1.137). No relationship was found between the Cdx2 and *BsmI* polymorphisms and the risk of PE (*p* < 0.05) [59].

Contrary to the results obtained in our research, the above analyses did not show a direct relationship between *BsmI* polymorphism and the increased risk of PE.

In the study of Baca et al., a population of women from the USA (744 preeclampsia cases and 2411 controls) was analyzed. The polymorphisms of three genes—*VDR*, *GC*, and *CYP27B1*—were analyzed. Minor alleles of the rs12831006 polymorphism in the non-coding region of the *VDR* gene were significantly correlated with the risk of PE (rs12831006: OR = 1.5, *p* < 0.0001). The study also showed a relationship between the polymorphic variants of the *GC* gene: one in the intron (rs843010: OR = 1.4, *p* < 0.05) and two variants in the flanking region (rs842991: OR = 1.5, *p* < 0.05; rs16846876: OR = 0.75, *p* < 0.05). An association of the *CYP27B1* gene polymorphism with the risk of PE was not found [60].

Some available analyses also suggest no influence of *VDR* gene polymorphisms on hypertensive complications in pregnancy. In the study by Rezende et al., the relationship between *VDR* polymorphisms (*FokI*, *ApaI*, and *BsmI*) and the occurrence of PE and GH (154 with GH, 162 with PE, and 213 healthy pregnant women) was analyzed. The frequency of all three *VDR* polymorphisms in the PE, GH, and healthy pregnant groups was similar (all *p* > 0.05) [61].

In our study we also performed haplotype analysis. Notably, each haplotype with significantly or borderline higher frequency in the PE group compared to controls contains the mutated *BsmI A* allele, which is correlated with PE development in our study (*CTA p* = 0.0241, *TAT p* = 0.0509, *TTAT p* = 0.0572).

Haplotype analysis has been performed in a few studies. Farajian-Mashhadi et al. reported that maternal and placental *TABf* haplotype may lead to decreased risk of PE development, and the placental *TABF* haplotype was associated with higher risk of PE [57].

Interesting results were also obtained in the analysis by Ghorbani et al., which indicated that in carriers of *CYP T*, *VDR T*, and *RXR A (TTA)* haplotypes compared to the GTG haplotype, the risk of PE was 6.71 times higher (*p* = 0.044). At the same time, in carriers of the heterozygous *VDR ApaI GT* genotype, an increased risk of PE (OR = 2.55, *p* = 0.04) was observed, while the presence of *VDR ApaI GT* + *TT* genotypes correlated with higher BMI and systolic blood pressure and a lower level of 25 (OH)-D3 [61].

In contrast to the above-mentioned literature, in a study by Rezende et al. haplotype frequency of *VDR* genes was similar in PE and GH groups and healthy pregnant women (all *p* > 0.05). These results did not show any association between *VDR* polymorphism or haplotypes and PE or GH development [60].

Caccamo et al. [62] analyzed *FokI* and *BsmI* polymorphisms of *VDR* gene in 116 women with gestational hypertension (GH) and 69 normotensive pregnant women. The authors reveal that *VDR FF/bB* haplotype is linked to GH development (increasing two folds GH risk). The interesting results show that insufficiency of vitamin D was detected in 92% GH women carrying of *FF/bB* haplotype. This study underlines that analysis of polymorphic variants could personalize vitamin D supplementation strategy in prevention of hypertensive disorders in pregnancy [62].

Additionally, in our study the correlation of *VDR BsmI* polymorphism with systolic and diastolic blood hypertension was noted. In carriers of the *BsmI A* allele, higher blood pressure values were observed. The results in our study reveal that the *VDR BsmI* polymorphism is closely associated with predisposition to higher hypertension. The results were corroborated by the research performed by Wang et al., in which the authors found associations of *VDR BsmI* and *FokI* variants with hypertension risk in Japanese men [49]. Additionally, a meta-analysis performed by Zhu et al. revealed a significant correlation between the *VDR BsmI* variant and susceptibility to hypertension [50]. The very exciting result in our study was the observation that 3-marker haplotype CTA (*TaqI, ApaI, BsmI*) was associated with significantly higher systolic (*p* = 0.0075) and diastolic (*p* = 0.0072) blood pressure values. This result indicates the combined influence of different polymorphisms on blood pressure modulation.

Summarizing the research carried out thus far, overall, it indicates the significant role of the endogenous vitamin D placental system in the development of PE. Furthermore, it was shown that VDR receptor is present in the decidua and syncytiotrophoblast, probably modulating the immune response and endothelial function. From this point of view, *VDR* polymorphic variants may play a crucial role in PE development. The results of our study provide additional evidence for the biological role of polymorphisms and haplotypes of the *VDR* gene in the etiology of PE. At this point, it should be emphasized that the analysis of polymorphic variants may lead to a personalized vitamin D supplementation strategy in the prevention of hypertensive disorders in pregnancy, which is a serious challenge in medical care.

Certainly, this study has a few limitations. First, a relatively small sized control group and low number of cases of early onset preeclampsia and late-onset preeclampsia. However, the analyzed group was selected very carefully with respect to age of patients and week of the end of pregnancy, allowing for a valuable case–control study analysis. On the other hand, the study covers the four most studied *VDR* polymorphisms. The frequency of the occurrence of particular genotypes and alleles was analyzed, and simultaneously haplotype analysis of the *VDR* gene polymorphisms was performed. Additionally, we carefully assessed the influence the *VDR* genetic variants on clinical parameters such as the blood pressure and body mass index of preeclamptic women. Another limitation is primiparity, which may be associated with elevated risk of preeclampsia. Moreover, in this study we did not perform measurements of the level of vitamin D in the study subjects. It should be emphasized that this kind of study indicating the importance of polymorphic variants of the *VDR* gene in PE etiology was performed for the first time in a population of preeclamptic Polish women. It makes this study valuable in the discussion about the pivotal role of VDR genetic variants in pathogenesis of hypertension in pregnancy in a particular European population and undoubtedly constitutes the strength of the study.

## 5. Conclusions

Association and haplotype analysis indicated that the *VDR BsmI A* allele could play a significant role in the PE pathomechanism and hence could be a risk factor for PE development. Additionally, the *VDR BsmI* polymorphism and CTA haplotype (TaqI/ApaI/BsmI), containing the *VDR BsmI A* allele, could be closely associated with higher values of systolic and diastolic blood pressure in the Polish population of pregnant women. However, the stratification analysis, after dividing PE group into early onset and late-onset form, showed also the influence of *VDR TaqI* and *FokI* polymorphisms on changing of blood pressure in pregnant women. Together the results of our study indicate the importance of the *VDR BsmI* polymorphism and reveal that this variant is closely associated with higher risk of PE development, thus these interesting results merit future studies.

## Figures and Tables

**Table 1:** Primary information of the selected VDR gene polymorphisms.

SNP	Chromosome Position *	Localization	Function	Alleles	Genomic Positions **
rs2228570 (*FokI*)	chr12:47879112	Exon 2	Met-Thr	*C>T*	g.30920*T>C*
rs1544410 (*BsmI*)	chr12:47846052	Intron 8	Non-coding	*G>A*	g.63980*G>A*
rs7975232 (*ApaI*)	chr12:47845054	Intron 8	Non-coding	*T>G*	g.64978*G>T*
rs731236 (*TaqI*)	chr12:47844974	Exon 9	Ile-Ile	*T>C*	g.65058*T>C*

* Location on chromosome based on human reference sequence (GRCh38.p12). ** Genomic positions refer to *VDR* reference sequence NG_008731.1.

**Table 2:** Clinical characteristics of the study population.

Variables	Preeclampsia n = 122	Controls n = 184	*p*
Maternal age (years), mean ± SD	30.12 ± 5.52	30.58 ± 4.41	0.4507
Gestational age (weeks), mean ± SD	33.72 ± 3.52	39.01 ± 1.21	<0.001
Systolic blood pressure (mmHg), mean ± SD	171.04 ± 19.27	102.31 ± 9.83	<0.001
Diastolic blood pressure (mmHg), mean ± SD	106.61 ± 12.89	64.97 ± 7,69	<0.001
Pre-pregnancy BMI (kg/m^2^), mean ± SD	24.56 ±.6.97	20.90 ± 1.97	<0.001
Post-pregnancy BMI (kg/m^2^), mean ± SD	30.40 ± 5.30	26.24 ± 2.60	<0.001
Caesarean section, N (%)	110 (90.16)	61 (33.15)	<0.001 *
Primipara, N (%)	75 (61.48)	72 (39.13)	0.0001 *
Infant birthweight (g), mean ± SD	1887.95 ± 841.64	3417.66 ± 428.15	<0.001
1-min Apgar score, median (IQR)	8.00 (6.00–9.75)	10.00 (10.00–10.00)	<0.001 #
5-min Apgar score, median (IQR)	9.00 (8.00–10.00)	10.00 (10.00–10.00)	<0.001 #

BMI = body mass index, *p* value—Student’s *t*-test, * Pearson’s χ^2^, # Mann–Whitney test.

**Table 3:** Characteristics of the case women according to onset of preeclampsia.

Variables	EOPE n = 57	LOPE n = 65	*p*
Maternal age (years), mean ± SD	29.70 ± 5.49	30.50 ± 5.57	0.4298
Gestational age (weeks), mean ± SD	30.60 ± 2.09	36.54 ± 1.69	<0.001
Systolic blood pressure (mmHg), mean ± SD	175.65 ± 19.51	166.97 ± 18.26	0.0171
Diastolic blood pressure (mmHg), mean ± SD	107.44 ± 13.53	105.88 ± 12.37	0.5268
Pre-pregnancy BMI (kg/m^2^), mean ± SD	24.46 ± 5.72	24.65 ± 7.93	0.8859
Post-pregnancy BMI (kg/m^2^), mean ± SD	30.02 ± 4.19	30.74 ± 6.12	0.4660
Caesarean section, N (%)	56 (98.25%)	52 (80.00%)	0.0171 **
Primipara, N (%)	35 (61.40%)	40 (61.54%)	0.9878 *
Infant birthweight (g), mean ± SD	1336.11 ± 433.69	2403.61 ± 803.82	<0.001
1-min Apgar score, median (IQR)	7.00 (4.00–8.00)	9.00 (8.00–10.00)	<0.001 #
5-min Apgar score, median (IQR)	8.00 (7.00–9.00)	10.00 (9.00–10.00)	<0.001 #
ALT (IU/L), mean ± SD	53.83 ± 58.51	23.08 ± 17.49	0.0411
AST (IU/L), mean ± SD	52.17 ± 52.50	25.50 ± 16.80	0.0492
Urea (mg/dL), mean ± SD	32.42 ± 13.65	29.59 ± 14.00	0.2740
Uremic acid (mg/dL), mean ± SD	6.74 ± 1.49	6.35 ± 1.16	0.1198
Total protein (g/dL), mean ± SD	5.47 ± 0.56	5.81± 0.78	0.0089
Creatinine (mg/dL), mean ± SD	0.81 ± 0.26	0.76 ± 0.30	0.5558
Proteinuria (mg/dL), mean ± SD	365.76 ± 185.91	195.07 ± 165.32	<0.001
Proteinuria (g/24 h), mean ± SD	4.57 ± 2.92	2.92 ± 3.64	0.1680

ALT = alanine transaminase; AST = aspartate transaminase, BMI = body mass index. *p* value—Student’s *t*-test, # Mann–Whitney test, * Pearson’s χ2, ** Fischer test.

**Table 4:** Prevalence of *VDR* alleles in PE cases (n = 244) and controls (n = 368).

SNP	Alleles	Preeclampsia n = 244		Controls n = 368		*p*	*p* Corr.
		MAF n (Frequency)	HWE *p*	MAF n (Frequency)	HWE *p*		
rs2228570 (*FokI*)	*C>T*	118 (0.484)	0.595	182 (0.495)	0.140	0.791	1.000
rs1544410 (*BsmI*)	*G>A*	114 (0.467)	0.021	130 (0.353)	0.104	0.005	0.020
rs7975232 (*ApaI*)	*T>G*	107 (0.439)	0.865	191 (0.519)	0.448	0.051	0.204
rs731236 (*TaqI*)	*T>C*	101 (0.414)	0.971	122 (0.332)	0.554	0.038	0.152

SNP—single-nucleotide polymorphism, MAF—minor allele frequency, HWE—Hardy–Weinberg equilibrium χ^2^, *p*—Pearson’s chi-squared test.

**Table 5:** Genotype distribution of the VDR gene polymorphisms in PE patients and healthy controls.

SNP	Genotypes/Models	Preeclampsia n = 122 n (%)	Controls n = 184 n (%)	OR (95%CI)	*p*	*p_adj._*
rs2228570 (*FokI*)	CC	34 (27.9)	42 (22.8)	1.00	0.3897	0.5040
	CT	58 (47.5)	102 (55.4)	1.42 (0.82–2.48)		
	TT	30 (24.6)	40 (21.7)	1.08 (0.56–2.08)		
	Dominant	88 (72.1)	142 (77.2)	1.31 (0.77–2.21)	0.3194	0.5383
	Recessive	92 (75.4)	144 (78.3)	0.85 (0.50–1.46)	0.5620	0.4498
rs1544410 (*BsmI*)	GG	41 (33.6)	82 (44.6)	1.00	0.0263	0.0113
	GA	48 (39.3)	74 (40.2)	0.77 (0.46–1.30)		
	AA	33 (27.0)	28 (15.2)	0.42 (0.23–0.79)		
	Dominant	81 (66.4)	102 (55.4)	0.63 (0.39–1.01)	0.0545	0.0334
	Recessive	89 (73.0)	156 (84.8)	0.48 (0.27–0.85)	0.0119	0.0048
rs7975232 (*ApaI*)	TT	38 (31.1)	40 (21.7)	1.00	0.1304	0.0765
	TG	61 (50.0)	97 (52.7)	1.51 (0.87–2.61)		
	GG	23 (18.9)	47 (25.5)	1.94 (1.00–3.79)		
	Dominant	84 (68.9)	144 (78.3)	1.63 (0.97–2.74)	0.0660	0.0425
	Recessive	99 (81.1)	137 (74.5)	1.48 (0.84–2.59)	0.1688	0.1064
rs731236 (*TaqI*)	TT	42 (34.4)	84 (45.7)	1.00	0.1171	0.0830
	TC	59 (48.4)	78 (42.4)	0.66 (0.40–1.09)		
	CC	21 (17.2)	22 (12.0)	0.52 (0.26–1.06)		
	Dominant	80 (65.6)	100 (54.3)	0.63 (0.39–1.00)	0.0498	0.0375
	Recessive	101 (82.8)	162 (88.0)	0.65 (0.34–1.25)	0.1987	0.1353

OR and 95%CI regression calculated, *p_adj._* values adjusted for BMI and primiparity.

**Table 6:** Genotypes distribution of VDR gene polymorphisms in EOPE and LOPE.

SNP	Genotypes/ Models	Controls (n = 184) n (%)	EOPE (n = 57) n (%)	OR (95%CI)	*p*	*p_adj._*	LOPE (n = 65) n (%)	OR (95%CI)	*p*	*p_adj._*
rs2228570 (*FokI*)	CC	42 (22.8)	14 (24.6)	1.00	0.5173	0.2164	20 (30.8)	1.00	0.4240	0.4995
	CT	102 (55.4)	27 (47.4)	1.26 (0.60–2.64)			31 (47.7)	1.57 (0.80–3.05)		
	TT	40 (21.7)	16 (28.1)	0.83 (0.36–1.93)			14 (21.5)	1.36 (0.61–3.05)		
	Dominant	142 (77.2)	43 (75.4)	1.10 (0.55–2.20)	0.7873	0.6852	45 (69.2)	1.50 (0.80–2.82)	0.2097	0.2387
	Recessive	144 (78.3)	41 (71.9)	0.71 (0.36–1.40)	0.3299	0.0815	51 (78.5)	1.01 (0.51–2.01)	0.9731	0.7277
rs1544410 (*BsmI*)	GG	82 (44.6)	17 (29.8)	1.00	0.0913	0.0464	24 (36.9)	1.00	0.0563	0.0835
	GA	74 (40.2)	26 (45.6)	0.59 (0.30–1.17)			22 (33.8)	0.98 (0.51–1.90)		
	AA	28 (15.2)	14 (24.6)	0.41 (0.18–0.95)			19 (29.2)	0.43 (0.21–0.90)		
	Dominant	102 (55.4)	40 (70.2)	0.53 (0.28–1.00)	0.0451	0.0498	41 (63.1)	0.73 (0.41–1.30)	0.2819	0.2465
	Recessive	89 (73.0)	43 (75.4)	0.55 (0.27–1.14)	0.1150	0.0305	46 (70.8)	0.43 (0.22–0.85)	0.0165	0.0263
rs7975232 (*ApaI*)	TT	40 (21.7)	17 (29.8)	1.00	0.0724	0.0356	21 (32.3)	1.00	0.2232	0.2956
	TG	97 (52.7)	33 (57.9)	1.25 (0.63–2.49)			28 (43.1)	1.82 (0.93–3.57)		
	GG	47 (25.5)	7 (12.3)	2.85 (1.08–7.57)			16 (24.6)	1.54 (0.71–3.35)		
	Dominant	144 (78.3)	40 (70.2)	1.53 (0.79–2.98)	0.2176	0.0911	44 (67.7)	1.72 (0.92–3.22)	0.0906	0.1258
	Recessive	137 (74.5)	50 (87.7)	2.45 (1.04–5.78)	0.0275	0.0185	49 (75.4)	1.05 (0.54–2.02)	0.8820	0.7935
rs731236 (*TaqI*)	TT	84 (45.7)	18 (31.6)	1.00	0.1613	0.1940	24 (36.9)	1.00	0.2311	0.2636
	TC	78 (42.4)	31 (54.4)	0.54 (0.28–1.04)			28 (43.1)	0.80 (0.43–1.49)		
	CC	22 (12.0)	8 (14.0)	0.59 (0.23–1.53)			13 (20.0)	0.48 (0.21–1.10)		
	Dominant	100 (54.3)	39 (68.4)	0.55 (0.29–1.03)	0.0574	0.0843	41 (63.1)	0.70 (0.39–1.25)	0.2199	0.2094
	Recessive	162 (88.0)	49 (86.0)	0.83 (0.35–1.99)	0.6815	0.2832	52 (80.0)	0.54 (0.26–1.15)	0.1200	0.1492

OR and 95%CI regression calculated, *p_adj_.* values adjusted for BMI and primiparity.

**Table 7:** Comparisons of quantitative traits of blood pressure between genotypes of *VDR* gene.

	Mean ± SD (mmHg)			Codominant		Dominant		Recessive	
	AA	AB	BB	*p*	*p_adj._*	*p*	*p_adj._*	*p*	*p_adj._*
rs2228570 (*FokI*)									
SBP	131.27 ± 35.25	124.91 ± 34.93	132.15 ± 39.84	0.2736	0.3156	0.3920	0.6805	0.3070	0.2073
DBP	81.87 ± 22.63	79.49 ± 22.15	81.90 ± 23.44	0.6620	0.6926	0.5860	0.9268	0.6048	0.4344
rs1544410 (*BsmI*)									
SBP	122.58 ± 33.55	127.69 ± 35.04	140.08 ± 41.00	0.0091	0.0033	0.0303	0.0172	0.0041	0.0015
DBP	76.74 ± 21.47	81.03 ± 21.51	87.57 ± 25.00	0.0094	0.0026	0.0148	0.0073	0.0074	0.0020
rs7975232 (*ApaI*)									
SBP	136.84 ± 40.02	126.79 ± 34.90	121.54 ± 33.16	0.0319	0.0121	0.0151	0.0066	0.0855	0.0436
DBP	85.34 ± 24.21	80.12 ± 22.00	76.54 ± 21.06	0.0591	0.0196	0.0345	0.0127	0.0870	0.0431
rs731236 (*TaqI*)									
SBP	122.56 ± 33.78	130.91 ± 36.45	135.71 ± 40.54	0.0641	0.0432	0.0263	0.0169	0.1451	0.1229
DBP	76.90 ± 21.93	82.54 ± 21.82	85.45 ± 25.15	0.0448	0.0263	0.0171	0.0104	0.1352	0.0936

SBP—systolic blood pressure, DBP—diastolic blood pressure, *p_adj._* values adjusted for BMI and primiparity. A—major allele, B—minor allele.

**Table 8:** Haplotype analysis of SNPs genotyped in the *VDR* gene.

rs731236	rs7975232	rs1544410	rs2228570	Frequency (Overall)	Frequency (PE, Control)	χ^2^	*p* Value	* *p* Value
T	G	G		0.476	0.425, 0.510	4.325	0.0376	0.1705
C	T	A		0.356	0.410, 0.320	5.090	0.0241	0.0940
T	T	G		0.117	0.104, 0.125	0.654	0.4185	0.9505
T	T	A		0.038	0.045, 0.033	0.657	0.4178	0.9503
	G	G	C	0.256	0.238, 0.269	0.734	0.3917	0.9553
	G	G	T	0.225	0.188, 0.250	3.225	0.0725	0.3043
	T	A	C	0.205	0.229, 0.190	1.347	0.2458	0.7768
	T	A	T	0.188	0.226, 0.163	3.812	0.0509	0.2231
	T	G	T	0.075	0.064, 0.082	0.708	0.4000	0.9594
	T	G	C	0.045	0.043, 0.046	0.028	0.8660	1.0000
T	G	G	C	0.252	0.236, 0.262	0.502	0.4789	0.9959
T	G	G	T	0.225	0.188, 0.249	3.091	0.0787	0.3742
C	T	A	C	0.190	0.220, 0.170	2.419	0.1199	0.5402
C	T	A	T	0.166	0.189, 0.150	1.598	0.2062	0.8185
T	T	G	T	0.073	0.062, 0.080	0.673	0.4122	0.9892
T	T	G	C	0.044	0.042, 0.045	0.046	0.8299	1.0000
T	T	A	T	0.022	0.036, 0.013	3.618	0.0572	0.2777
T	T	A	C	0.016	0.009, 0.020	1.015	0.3138	0.9720

* *p* value calculated using permutation test and a total of 10,000 permutations.

**Table 9:** Estimated frequency of haplotypes and association with risk of higher blood pressure.

4-Marker		SBP			DBP		
Haplotype	Frequency	Difference	95%CI	*p*-Value	Difference	95%CI	*p*-Value
TGGC	0.253	122.07	Ref.		76.31	Ref.	
CTAC	0.195	7.94	(−2.24–18.13)	0.1264	6.10	(−0.37–12.57)	0.0648
CTAT	0.162	8.12	(−1.55–17.79)	0.0998	4.57	(−1.42–10.56)	0.1348
TTGT	0.073	2.63	(−10.61–15.86)	0.6970	0.90	(−7.42–9.21)	0.8324
TGGT	0.222	-2.17	(−11.79–7.45)	0.6587	−0.51	(−6.72–5.71)	0.8728
rare	0.094	4.99	(−6.38–16.37)	0.3897	3.00	(−4.29–10.28)	0.4201
3-marker							
TGG	0.475	120.21	Ref.		75.93	Ref.	
CTA	0.357	8.84	(2.36–15.33)	0.0075	5.53	(1.50–9.56)	0.0072
TTG	0.114	3.59	(−6.10–13.27)	0.4677	1.38	(−4.64–7.40)	0.6529
rare	0.053	7.76	(−4.23–19.75)	0.2046	4.12	(−3.33–11.58)	0.2780

(*TaqI/ApaI/BsmI/FokI*).

## References

[1] Hutcheon J.A., Lisonkova S., Joseph K.S. (2011). Epidemiology of pre-eclampsia and the other hypertensive disorders of pregnancy. Best Pract. Res. Clin. Obstet. Gynaecol..

[2] Teliga-Czajkowska J., Czajkowski K., Januszewicz A., Sieradzki J., Więcek A., Szczeklik A., Gajewski P. (2009). Nadciśnienie tętnicze i diabetologia w pytaniach i odpowiedziach. Nadciśnienie Tętnicze a Ciąża.

[3] Szczepaniak-Chicheł L., Bręborowicz G., Tykarski A. (2006). Leczenie nadciśnienia tętniczego u kobiet w ciąży. Nadciśnienie Tętnicze.

[4] Bokslag A., van Weissenbruch M., Mol B.W., de Groot C.J.M. (2016). Preeclampsia; short and long-term consequences for mother and neonate. Early Hum. Dev..

[5] McKinney D., Boyd H., Langager A., Oswald M., Pfister A., Warshak C.R. (2016). The impact of fetal growth restriction on latency in the setting of expectant management of preeclampsia. Am. J. Obstet. Gynecol..

[6] Fox R., Kitt J., Leeson P., Aye C.Y.L., Lewandowski A.J. (2019). Preeclampsia: Risk factors, diagnosis, management and the cardiovascular impact on the offspring. J. Clin. Med..

[7] Ożarowski M., Karpiński T.M., Szulc M., Wielgus K., Kujawski R., Wolski H., Seremak-Mrozikiewicz A. (2021). Plant phenolics and extracts in animal models of preeclampsia and clinical trials—Review of perspectives for novel therapies. Pharmaceuticals.

[8] Knabl J., Vattai A., Ye Y., Jueckstock J., Hutter S., Kainer F., Mahner S., Jeschke U. (2017). Role of placental VDR expression and function in common late pregnancy disorders. Int. J. Mol. Sci..

[9] Rana S., Lemoine E., Granger J.P., Karumanchi S.A. (2019). Preeclampsia: Pathophysiology, challenges, and perspectives. Circ Res..

[10] Jena M.K., Sharma N.R., Petitt M., Maulik D., Nayak N.R. (2020). Pathogenesis of preeclampsia and therapeutic approaches targeting the placenta. Biomolecules.

[11] Tsuprykov O., Chen X., Hocher C.F., Skoblo R., Lianghong Y., Hocher B. (2018). Why should we measure free 25(OH) vitamin D?. J. Steroid Biochem. Mol. Biol..

[12] Hollis B.W. (2008). Assessment of vitamin D status and definition of normal circulating range of 25-hydroxyvitamin D. Curr. Opin. Endocrinol. Diabetes. Obes..

[13] Holick M.F. (2008). Vitamin D: A D-lightful health perspective. Nutr. Rev..

[14] Lensmeyer G., Poquette M., Wiebe D., Binkley N. (2012). The C-3 epimer of 25-hydroxyvitamin D(3) is present in adult serum. J. Clin. Endocrinol. Metab..

[15] Specker B. (2004). Vitamin D requirements during pregnancy. Am. J. Clin. Nutr..

[16] Williams A.F. (2007). Vitamin D In pregnancy: An old problem still to be solved?. Arch. Dis. Child..

[17] Hollis B.W., Wagner C.L. (2013). Vitamin D and pregnancy: Skeletal effects, nonskeletal effects, and birth outcomes. Calcif. Tissue Int..

[18] Colonese F., Laganà A.S., Colonese E., Sofo V., Salmeri F.M., Granese R., Triolo O. (2015). The pleiotropic effects of vitamin D in gynaecological and obstetric diseases: An overview on a hot topic. Biomed. Res. Int..

[19] Wagner C.L., Hollis B.W. (2018). The Implications of Vitamin D Status during Pregnancy on Mother and her Developing Child. Front. Endocrinol..

[20] Powe C.E., Levine R.J., Karumanchi S.A. (2011). Preeclampsia, a disease of the maternal endothelium: The role of antiangiogenic factors and implications for later cardiovascular disease. Circulation.

[21] Widmer R.J., Lerman A. (2014). Endothelial dysfunction and cardiovascular disease. Glob. Cardiol. Sci. Pract..

[22] Konukoglu D., Uzun H. (2016). Endothelial dysfunction and hypertension. Adv. Exp. Med. Biol..

[23] Liberis A., Stanulov G., Ali E.C., Hassan A., Pagalos A., Kontomanolis E.N. (2016). Pre-eclampsia and the vascular endothelial growth factor: A new aspect. Clin. Exp. Obstet. Gynecol..

[24] Guney G., Taskin M.I., Tokmak A. (2020). Increase of circulating inflammatory molecules in preeclampsia, an update. Eur. Cytokine Netw..

[25] Harmon A.C., Cornelius D.C., Amaral L.M., Faulkner J.L., Cunningham M.W., Wallace K., LaMarca B. (2016). The role of inflammation in the pathology of preeclampsia. Clin. Sci..

[26] Barrera D., Díaz L., Noyola-Martínez N., Halhali A. (2015). Vitamin D and inflammatory cytokines in healthy and preeclamptic pregnancies. Nutrients.

[27] Chan S.Y., Susarla R., Canovas D., Vasilopoulou E., Ohizua O., McCabe C.J., Hewison M., Kilby M.D. (2015). Vitamin D promotes human extravillous trophoblast invasion in vitro. Placenta.

[28] Du H., Daftary G.S., Lalwani S.I., Taylor H.S. (2005). Direct regulation of HOXA10 by 1,25-(OH)2D3 in human myelomonocytic cells and human endometrial stromal cells. Mol. Endocrinol..

[29] Boonstra A., Barrat F.J., Crain C., Heath V.L., Savelkoul H.F., O’Garra A. (2001). 1alpha,25-Dihydroxyvitamin d3 has a direct effect on naive CD4(+) T cells to enhance the development of Th2 cells. J. Immunol..

[30] Cardús A., Parisi E., Gallego C., Aldea M., Fernández E., Valdivielso J.M. (2006). 1,25-Dihydroxyvitamin D3 stimulates vascular smooth muscle cell proliferation through a VEGF-mediated pathway. Kidney Int..

[31] Barrera D., Noyola-Martínez N., Avila E., Halhali A., Larrea F., Díaz L. (2012). Calcitriol inhibits interleukin-10 expression in cultured human trophoblasts under normal and inflammatory conditions. Cytokine.

[32] Xu J., Gu Y., Lewis D.F., Cooper D.B., McCathran C.E., Wang Y. (2019). Downregulation of vitamin D receptor and miR-126-3p expression contributes to increased endothelial inflammatory response in preeclampsia. Am. J. Reprod. Immunol..

[33] Li Y.C. (2003). Vitamin D regulation of the renin-angiotensin system. J. Cell Biochem..

[34] Akbari S., Khodadadi B., Ahmadi S.A.Y., Abbaszadeh S., Shahsavar F. (2018). Association of vitamin D level and vitamin D deficiency with risk of preeclampsia: A systematic review and updated meta-analysis. Taiwan J. Obstet. Gynecol..

[35] Lumbers E.R., Delforce S.J., Arthurs A.L., Pringle K.G. (2019). Causes and Consequences of the Dysregulated Maternal Renin-Angiotensin System in Preeclampsia. Front. Endocrinol..

[36] Baker A.R., McDonnellm D.P., Hughes M., Crisp T.M., Mangelsdorf D.J., Haussler M.R., Pike J.W., Shine J., O’Malley B.W. (1988). Cloning and expression of full-length cDNA encoding human vitamin D receptor. Proc. Natl. Acad. Sci. USA.

[37] Uitterlinden A.G., Fang Y., Van Meurs J.B.J., Pols H.A.P., Van Leeuwen J.P. (2004). Genetics and biology of vitamin D receptor polymorphisms. Gene.

[38] Arai H., Miyamoto K., Taketani Y., Yamamoto H., Iemori Y., Morita K., Tonai T., Nishisho T., Mori S., Takeda E. (1997). A vitamin D receptor gene polymorphism in the translation initiation codon: Effect on protein activity and relation to bone mineral density in Japanese women. J. Bone Miner. Res..

[39] (2019). ACOG. ACOG practice bulletin no. 202: Gestational hypertension and preeclampsia. Obs. Gynecol.

[40] Farajian-Mashhadi F., Eskandari F., Rezaei M., Eskandari F., Najafi D., Teimoori B., Moradi-Sharbabak M., Salimi S. (2020). The possible role of maternal and placental vitamin D receptor polymorphisms and haplotypes in pathogenesis of preeclampsia. Clin. Exp. Hypertens..

[41] Barchitta M., Maugeri A., La Rosa M.C., San Lio R.M., Favara G., Panella M., Cianci A., Agodi A. (2018). The effect of vitamin D receptor gene (VDR) polymorphisms on adverse pregnancy outcomes—Including preterm birth (PTB and small for gestational age. Nutrients.

[42] Pani M.A., Knapp M., Donner H., Braun J., Baur M.P., Usadel K.H., Badenhoop K. (2000). Vitamin D receptor allele combinations influence genetic susceptibility to type 1 diabetes in Germans. Diabetes.

[43] Morrison N.A., Qi J.C., Tokita A., Kelly P.J., Crofts L., Nguyen T.V., Sambrook P.N., Eisman J.A. (1994). Prediction of bone density from vitamin D receptor alleles. Nature.

[44] Harris S.S., Eccleshall T.R., Gross C., Dawson-Hughes B., Feldman D. (1997). The vitamin D receptor start codon polymorphism (FokI) and bone mineral density in premenopausal American black and white women. J. Bone Miner. Res..

[45] R Core Team (2019). A Language and Environment for Statistical Computing.

[46] González J.R., Armengol L., Solé X., Guinó E., Mercader J.M., Estivill X., Moreno V. (2007). SNPassoc: An R package to perform whole genome association studies. Bioinformatics.

[47] Wang L., Ma J., Manson J.E., Buring J.E., Gaziano J.M., Sesso H.D. (2013). A prospective study of plasma vitamin D metabolites, vitamin D receptor gene polymorphisms, and risk of hypertension in men. Eur. J. Nutr..

[48] Valdivielso J.M., Fernandez E. (2006). Vitamin D receptor polymorphisms and diseases. Clin. Chim. Acta.

[49] Zhu Y.B., Li Z.Q., Ding N., Yi H.L. (2019). The association between vitamin D receptor gene polymorphism and susceptibility to hypertension: A meta-analysis. Eur. Rev. Med. Pharmacol. Sci..

[50] Liu J., Dai Q., Li W., Guo Y., Dai A., Wang Y., Deng M., Tang Z., She L., Chen X. (2021). Association of vitamin D receptor gene polymorphisms with gestational diabetes mellitus-a case control study in Wuhan, China. BMC Pregnancy Childbirth.

[51] Zhou Q., Wen S., Liu M., Zhang S., Jin X., Liu A. (2020). Association between Gene Polymorphisms of Vitamin D Receptor and Gestational Diabetes Mellitus: A Systematic Review and Meta-Analysis. Int. J. Environ. Res. Public Health.

[52] Apaydın M., Beysel S., Eyerci N., Pinarli F.A., Ulubay M., Kizilgul M., Ozdemir O., Caliskan M., Cakal E. (2019). The VDR gene FokI polymorphism is associated with gestational diabetes mellitus in Turkish women. BMC Med. Genet..

[53] Krpina M.G., Barišić A., Peterlin A., Tul N., Ostojić S., Peterlin B., Pereza N. (2020). Vitamin D receptor polymorphisms in spontaneous preterm birth: A case-control study. Croat. Med. J..

[54] Barišić A., Pereza N., Hodžić A., Krpina M.G., Ostojić S., Peterlin B. (2021). Genetic variation in the maternal vitamin D receptor FokI gene as a risk factor for recurrent pregnancy loss. J. Matern. Fetal. Neonatal. Med..

[55] Wolski H., Kurzawińska G., Ożarowski M., Mrozikiewicz A.E., Drews K., Karpiński T.M., Bogacz A., Seremak-Mrozikiewicz A. (2021). Vitamin D receptor gene polymorphisms and haplotypes in the etiology of recurrent miscarriages. Sci. Rep..

[56] Wolski H., Kurzawinska G., Ozarowski M., Drews K., Barlik M., Piatek K., Malewski Z., Mrozikiewicz A.E., Magielda-Stola J., Kolanowska D. (2020). FokI vitamin D receptor polymorphism as a protective factor in intrahepatic cholestasis of pregnancy. Ginekol. Pol..

[57] Rezavand N., Tabarok S., Rahimi Z., Vaisi-Raygani A., Mohammadi E., Rahimi Z. (2019). The effect of VDR gene polymorphisms and vitamin D level on blood pressure, risk of preeclampsia, gestational age, and body mass index. J. Cell. Biochem..

[58] Zhan Y., Liu M., You Y., Zhang Y., Wang J., Wang X., Liu S., Liu X. (2015). Genetic variations in the vitamin-D receptor (VDR) gene in preeclampsia patients in the Chinese Han population. Hypertens. Res..

[59] Baca K.M., Govil M., Zmuda J.M., Simhan H.N., Marazita M.L., Bodnar L.M. (2018). Vitamin D metabolic loci and preeclampsia risk in multi-ethnic pregnant women. Physiol. Rep..

[60] Rezende V.B., Sandrim V.C., Palei A.C., Machado L., Cavalli R.C., Duarte G., Tanus-Santos J.E. (2012). Vitamin D receptor polymorphisms in hypertensive disorders of pregnancy. Mol. Biol. Rep..

[61] Ghorbani Z., Shakiba M., Rezavand N., Rahimi Z., Vaisi-Raygani A., Rahimi Z., Shakiba E. (2021). Gene variants and haplotypes of Vitamin D biosynthesis, transport, and function in preeclampsia. Hypertens. Pregnancy.

[62] Caccamo D., Cannata A., Ricca S., Catalano L.M., Montalto A.F., Alibrandi A., Ercoli A., Granese R. (2020). Role of Vitamin-D Receptor (VDR) single nucleotide polymorphisms in gestational hypertension development: A case-control study. PLoS ONE..

